# Editorial: Magnetobiology and chronobiology: new opportunities for smart phytoprotection

**DOI:** 10.3389/fpls.2025.1594646

**Published:** 2025-04-01

**Authors:** Guijun Wan, Gregory A. Sword, Juan Du, Qiuying Huang, Wenfeng Chen, Eric Warrant

**Affiliations:** ^1^ State Key Laboratory of Agricultural and Forestry Biosecurity, College of Plant Protection, Nanjing Agricultural University, Nanjing, China; ^2^ Department of Entomology, Texas A&M University, College Station, United States; ^3^ State Key Laboratory of Agricultural and Forestry Biosecurity, MOA Key Lab of Pest Monitoring and Green Management, College of Plant Protection, China Agricultural University, Beijing, China; ^4^ Hubei Insect Resources Utilization and Sustainable Pest Management Key Laboratory, Huazhong Agricultural University, Wuhan, China; ^5^ Institute of Life Sciences, College of Biological Science and Engineering, Fuzhou University, Fuzhou, China; ^6^ Lund Vision Group, Department of Biology, Lund University, Lund, Sweden

**Keywords:** magnetobiology, chronobiology, smart phytoprotection, crop protection, magnetic fields, biological rhythms, precision agriculture, ecological safety

## 1 Introduction

Smart Phytoprotection is an innovative interdisciplinary field integrating plant sciences with advanced technologies, aimed at developing sustainable, precise, and responsive solutions to protect crops against environmental and biotic stressors (Huang and Shu, 2021). Two rapidly advancing but relatively underutilized disciplines, magnetobiology (Binhi and Rubin, 2022; Zhang, 2023) and chronobiology (Dunlap et al., 2004; Numata and Tomioka, 2023), offer unique opportunities for more effective and ecologically sound plant protection strategies. Magnetobiology investigates the effects of magnetic fields on living organisms, while Chronobiology studies biological rhythms and their responses to environmental factors. The magnetoresponse of the circadian clock has been independently identified by multiple research groups (Krylov et al., 2022; Fedele et al., 2014; Yoshii et al., 2009), suggesting a fundamental interplay between magnetoreception and circadian mechanisms. Furthermore, the widespread conservation of both magnetoresponse (Zhang, 2023; Lin et al., 2020) and circadian regulation (Dunlap et al., 2004; Dunlap, 1999) across diverse taxa highlights their evolutionary and biological significance. Over the past two decades, the fields of magnetobiology and chronobiology have advanced considerably, opening new avenues for interdisciplinary applications. However, their integration into Smart Phytoprotection remains largely unexplored, presenting potential for new innovations in this field.

This Research Topic presents insights from Magnetobiology, Chronobiology, and Artificial Intelligence (AI), offering inspiration for their potential role in developing sustainable approaches to crop protection.

## 2 Magnetobiology: enhancing crop resilience and ensuring genomic safety

Zhou et al. demonstrated beneficial effects of moderate static magnetic fields (SMF) on *Arabidopsis thaliana*, improving plant growth and stress tolerance. This work suggests moderate SMF is involved in regulating the growth and development of *Arabidopsis thaliana* through maintaining iron homeostasis and balancing oxidative stress, which could be beneficial for plant survival and growth. Understanding the mechanisms behind magnetic field effects on plants and their associated regulatory networks could provide valuable insights for developing novel plant synthetic biology technologies, enabling the engineering of stress-resistant and high-yielding crops. For Smart Phytoprotection, the ability to modulate plant physiology using magnetic fields offers exciting possibilities. Magnetic treatment could be integrated into precision agricultural systems to optimize plant development, and improve resistance to abiotic stresses. Xu et al. provided critical insights into genomic safety concerning ultra-high static magnetic fields (UHSMF). Their research reported stable overall mutation rates yet identified subtle genomic alterations, such as decreased nucleotide transition rates and increased frequencies of larger insertions and deletions. These findings highlight the importance of rigorous genomic monitoring when considering the agricultural application of technologies involving UHSMF. Understanding the genomic effects of prolonged magnetic exposure is essential for ensuring the safety and stability of crop genomes.

## 3 Chronobiology: timing as a key for pest management

Miller et al. observed that allochronic behavior in field populations of the fall armyworm, *Spodoptera frugiperda*, aligns with previous laboratory findings on mating timing differences between strains. However, they also noted increased variability in behavior within and across native populations, posing challenges for predictive models that use pheromone trap capture timing as a phenotypic marker for strain identification. These findings underscore the importance of integrating circadian rhythms into Smart Phytoprotection, as variation in the timing of pest behavior can be used to enhance the precision of monitoring and management strategies. For example, aligning pesticide applications or pheromone trap monitoring times with the active periods of specific pest strains could enhance management efficiency while minimizing chemical use and ecological impact.

## 4 Driving technological advancements in smart agriculture

Gong et al. proposed an advanced version of the YOLOX-Tiny model optimized for maize crop row navigation line recognition. Incorporating adaptive illumination adjustment, multi-scale prediction, and attention mechanisms, their method enhanced detection accuracy and operational efficiency. Such AI-driven methodologies hold great promise for increasing agricultural productivity while minimizing chemical herbicide usage. The integration of AI with Magnetobiology and Chronobiology, and AI remains a promising yet open question in Smart Phytoprotection.

## 5 Future perspectives and challenges

The integration of Magnetobiology, Chronobiology, and AI offers promising opportunities for smarter, ecologically sound agricultural practices, but also presents specific challenges: a) Mechanistic Understanding: The molecular and physiological mechanisms underlying chronobiological and magnetoresponses in plants and pests remain poorly understood. Further research is required to elucidate the pathways linking magnetic fields, circadian rhythms, and phytoprotection. b) Field Application and Scalability: Many findings in Magnetobiology and Chronobiology have been established under controlled laboratory conditions. Translating these insights into field applications requires testing across diverse environmental conditions, crop species, and pest populations. c) Technological Integration: Smart Phytoprotection relies on sensor technologies, AI-driven models, and automated control systems. Future research should explore how magnetic and chronobiological factors can be integrated into real-time monitoring and precision intervention systems. d) Ecological and Safety Considerations: The long-term effects of magnetic exposure on ecosystems, soil microbiomes, and non-target organisms remain largely unexplored. Comprehensive risk assessments will be crucial to ensure that Smart Phytoprotection strategies do not inadvertently disrupt natural ecological balances.

## 6 Conclusion

This Research Topic called for further exploration of the emerging role of magnetobiology and chronobiology in Smart Phytoprotection, encouraging new discoveries that could enhance pest management, optimize plant growth, and promote environmental safety. The collected studies provide valuable insights, encouraging new interdisciplinary approaches to advance sustainable agriculture. By addressing key knowledge gaps and embracing innovative technologies, researchers can unlock the full potential of Smart Phytoprotection, ultimately contributing to more efficient, resilient, and sustainable agricultural systems.

We sincerely thank all contributing authors for their valuable research and hope these insights will inspire further advancements in Smart Phytoprotection.
